# Effects of postoperative adjuvant steroid therapy on the outcomes of biliary atresia: A systematic review and updated meta-analysis

**DOI:** 10.3389/fphar.2022.956093

**Published:** 2022-09-14

**Authors:** Chang-zhen Yang, Yan Zhou, Meng Ke, Ru-yue Gao, Shi-ru Ye, Mei Diao, Long Li

**Affiliations:** ^1^ Department of Pediatric Surgery, Capital Institute of Pediatrics, Beijing, China; ^2^ Graduate School of Peking Union Medical College, Chinese Academy of Medical Sciences, Beijing, China; ^3^ Research Unit of Minimally Invasive Pediatric Surgery on Diagnosis and Treatment(2021RU015), Chinese Academy of Medical Sciences, Beijing, China

**Keywords:** biliary atresia, kasai portoenterostomy, steroid treatment, outcomes, meta-analysis

## Abstract

**Background:** Postoperative adjuvant steroid therapy is regarded as the conventional treatment for patients with biliary atresia (BA) who have undergone Kasai portoenterostomy (KP). However, whether the steroid therapy can improve BA outcomes is controversial. This meta-analysis aimed to evaluate the effects of adjuvant steroid therapy on the surgical prognosis of BA.

**Methods:** We searched related studies published in PubMed, Embase, Web of Science, Cochrane, and the Chinese National Knowledge Infrastructure database up to May 2022. Data on the effect of steroid use on the clinical prognosis of the patients, including the jaundice clearance rate (JCR), native liver survival rate (NLSR) at 6, 12, and 24 months after KP, and the incidence of cholangitis, were extracted. Subgroup analyses based on age at KP, administration method, initial dosage, and steroid type were conducted. Statistical analysis was conducted using Stata/SE 12.0.

**Results:** Eleven articles (a total of 1,032 patients) were included in the present meta-analysis. The results demonstrated that postoperative adjuvant steroid therapy improved JCR at the 6/12/24-month follow-up (RR: 1.35, 95% CI: 1.18–1.55, *p* < 0.001; RR:1.49, 95% CI, 1.12–1.99, *p* = 0.006; RR: 1.41, 95% CI: 1.14–1.75, *p* = 0.002) and improved NLSR at the 24-month follow-up (RR: 1.31, 95% CI: 1.03–1.68, *p* = 0.028). However, steroids could not significantly improve NLSR at the 6/12-month follow-up (RR: 1.06; 95% CI: 0.98–1.15; *p* = 0.17; RR: 1.22; 95% CI: 0.97–1.54; *p* = 0.095), and might not decrease the incidence of postoperative cholangitis (RR: 0.78, 95% CI: 0.60–1.01, *p* = 0.058). Furthermore, subgroup analyses confirmed that three variables (age at KP, administration method, and initial dosage) could affect the efficacy of steroids in BA patients.

**Conclusion:** Postoperative adjuvant steroid therapy can significantly improve bile flow. The superiority of steroid therapy was more remarkable in patients aged ≤70 days at KP than in those aged >70 days. Additionally, intravenous followed by oral steroid administration method and medium initial dosage seemed to have the more reliable efficiency on bile flow. And patients treated by steroid had better long-term (24-month) native liver survival, but there is no significant effect on short-term native liver survival and postoperative cholangitis. Further studies are warranted.

## Introduction

Biliary atresia (BA) is a serious neonatal disease caused by intrahepatic or extrahepatic bile duct occlusions. The incidence of BA ranges from 1 in 5000 to 1 in 19,000 live births (Chardot, 2006; Townsend et al., 2018). Kasai portoenterostomy (KP) is currently considered the primary procedure for restoring bile flow and preserving the native liver in patients with BA (Davenport, 2012; Shinkai et al., 2009; Pakarinen and Rintala, 2011; Zhang et al., 2014). However, even after a successful KP, most patients still require liver transplantation before adulthood (Davenport, 2012; Kelay and Davenport, 2017). Hence, it is important for patients with BA to receive rational postoperative adjuvant therapy following KP. Based on its potential ability to regulate bile acid metabolism and its anti-inflammatory (Dillon et al., 2001; Sokol, 2007) and immunomodulatory effects, steroid therapy, which is widely used by many centers, has been regarded a conventional postoperative adjuvant treatment (Sokol, 2007; Muraji et al., 2004). However, the complex pharmacological mechanism of steroids has not been elucidated, resulting in uncertainties regarding its clinical efficacy for BA patients. Hence, many centers have conducted studies on the effect of postoperative steroids on the outcomes of BA. Several retrospective studies have shown that steroid therapy can promote bile flow and native liver survival (Dillon et al., 2001; Meyers et al., 2003; Kobayashi et al., 2005). Furthermore, a newly published report of a randomized controlled trial (RCT) by Lu et al. concluded that postoperative steroid therapy can significantly improve jaundice clearance rate (JCR) and native liver survival rate (NLSR) in patients with type III BA who have undergone KP (Lu et al., 2022). However, some studies have demonstrated that patients with BA could not derive certain benefits from postoperative steroid therapy (Davenport et al., 2007; Petersen et al., 2008; Davenport et al., 2013). Because of the inconsistencies in the findings of previous studies, the effect of postoperative steroid treatment on the outcomes of patients with BA is debatable.

In some previous systematic reviews and meta-analyses (Zhang et al., 2014; Chen et al., 2015), there was no evidence supporting that steroids could improve bile flow and native liver survival and decrease the incidence of postoperative cholangitis. A systematic review and meta-analysis conducted by Zhang in 2016 demonstrated that steroid therapy could improve JCR for only up to 1 year and was unable to improve NLSR (Zhang S et al., 2017). Furthermore, limited by their sample size, these reviews failed to obtain definitive conclusions or include subgroup analyses. In the present meta-analysis, we incorporated all relevant studies and aimed to elucidate the effects of steroid therapy on bile flow, native liver survival, and postoperative cholangitis. We also aimed to explore the potential factors that could affect the efficiency of steroids.

## Methods

### Search strategy

The present systematic review and meta-analysis was conducted based on the guidelines of Preferred Instrument for Systematic Reviews and Meta-Analysis (PRISMA) (Knobloch et al., 2011). *A priori* protocol was followed and registered in PROSPERO (CRD42022335999). The PICO (Participants/Intervention/Comparison/Outcome) framework was used as follows. Participant was BA patients who have undergone KP, the intervention was postoperative steroid therapy, the comparison was placebo or no steroid groups, and outcomes were native liver rate, jaundice clearance rate, and incidence of cholangitis. We performed a systematic search of PubMed, Embase, Web of Science, Cochrane, and Chinese National Knowledge Infrastructure database up to May 2022. The search terms included the following: “Portoenterostomy, Hepatic”, “Biliary Atresia”, “Steroids/Adrenal Cortex Hormones/Glucocorticoids”. The detailed search strategy can be found in Supplementary Table S2. In addition, a manual search of reference lists of relevant articles was also conducted, aiming to identify additional relevant studies.

### Eligibility criteria and data extraction

We incorporated all related studies. The inclusion criteria were as follows: (I) original studies published in English or in other languages, with an English abstract; (II) RCTs or cohort studies comparing the effects of steroids vs*.* non-steroids in BA patients who have undergone KP; and (III) studies reporting outcome indicators such as incidence of postoperative cholangitis, JCR, or NLSR at 6/12/24 months. Duplicate publications, low-quality articles, animal studies, and nonoriginal researches were excluded. The following information were independently extracted by two researchers (YCZ and ZY): First author, publication year, country of study, study design, sample size, patient age at KP, total bilirubin level at KP, type of BA, steroid regimen, other adjuvant therapies, and clinical outcome indicators.

### Quality assessment and publication bias

Two independent authors (YCZ and KM) rated the quality of the included cohort studies using the Newcastle–Ottawa Scale (NOS) for cohort studies. Studies with NOS scores of ≥6 were included in the meta-analysis. The quality of each included RCT was independently evaluated according to the Cochrane Handbook for Systematic Reviews of Interventions (Vader, 1998). Patient selection and allocation, blinding methods, data completeness, and other biases were evaluated. All assessment results were divided into “low risk of bias (+)”, “high risk of bias (−)”, and “moderate risk of bias (?)”. In addition, publication bias was assessed by using Egger’s test. Statistical significance was set at *p* < 0.05.

### Statistical methods

Statistical analyses were conducted by using Stata/SE 12.0. Relative risk (RR) and 95% confidence interval (CI) were calculated. Statistical significance was set at *p* < 0.05. We conducted our meta-analysis using the random effects model according to the diversity of patients, study designs and outcomes. Furthermore, subgroup analyses were performed to explore factors associated with the efficiency of steroid on patients with BA, including age at KP, steroid regimen, and study design. First, we conducted a subgroup analysis based on operative age stratification (mean or median age ≤70 days vs*.* > 70 days). Second, subgroup analyses were performed according to drug administration methods, initial dosage, and steroid type. Administration methods were divided into intravenous (IV), oral, IV followed by oral, and rectally-administered method. In the initial dosage stratification, the doses of various glucocorticoids were converted to an equivalent dose of methylprednisolone. High dosage was defined as ≥10 mg/kg/d methylprednisolone, “medium initial dosage” was defined as 4 mg/kg/d methylprednisolone, and low initial dosage was defined as 1–2 mg/kg/d methylprednisolone. Furthermore, patients were subdivided, according to steroid type, into methylprednisolone, prednisone, and methylprednisolone and prednisone. A subgroup analysis of study design was also performed by dividing studies into RCTs and cohort studies. Additionally, a sensitivity analysis was conducted to identify the reliability and validity of the analyses by observing the pooled RR after removing each study.

## Results

### Search results and study characteristics

**FIGURE 1:**
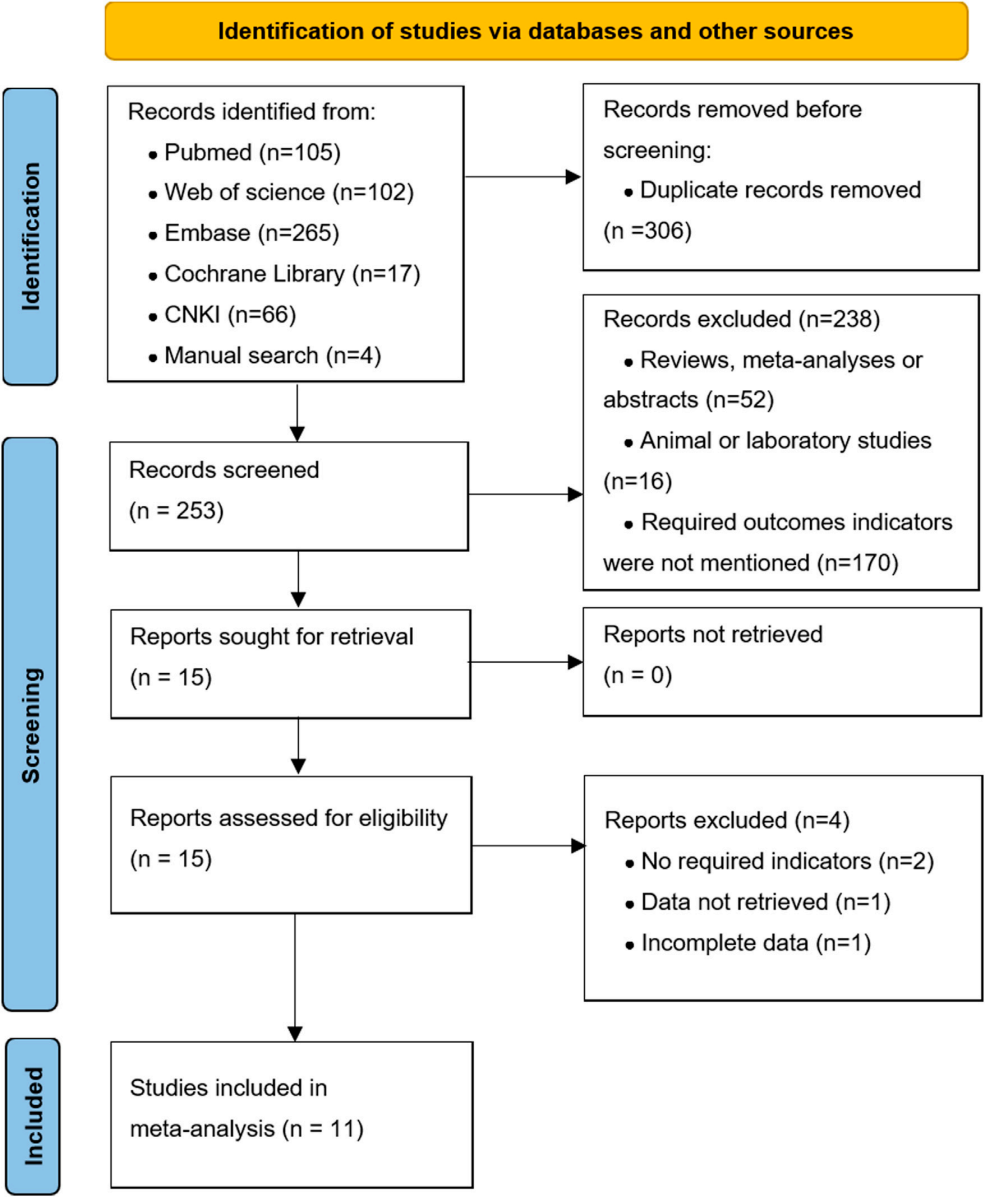
Flowchart on literature search and study selection.

Figure 1 shows the literature search and screening process. We obtained 559 articles from databases (*n* = 555) and manual searches (*n* = 4). Of these, 306 articles were excluded because of duplication. After reviewing the titles and abstracts, there were 15 articles for further full-text screened. Finally, our meta-analysis included 11 high-quality articles (Meyers et al., 2003; Escobar et al., 2006; Davenport et al., 2007; Vejchapipat et al., 2007; Chung et al., 2008; Petersen et al., 2008; Davenport et al., 2013; Bezerra et al., 2014; Yue et al., 2016; Kuebler et al., 2021; Lu et al., 2022) (1,032 patients), including five RCTs and six cohort studies. The characteristics of the included studies are shown in Supplementary Data Sheet S1.

### Quality assessment

**FIGURE 2:**
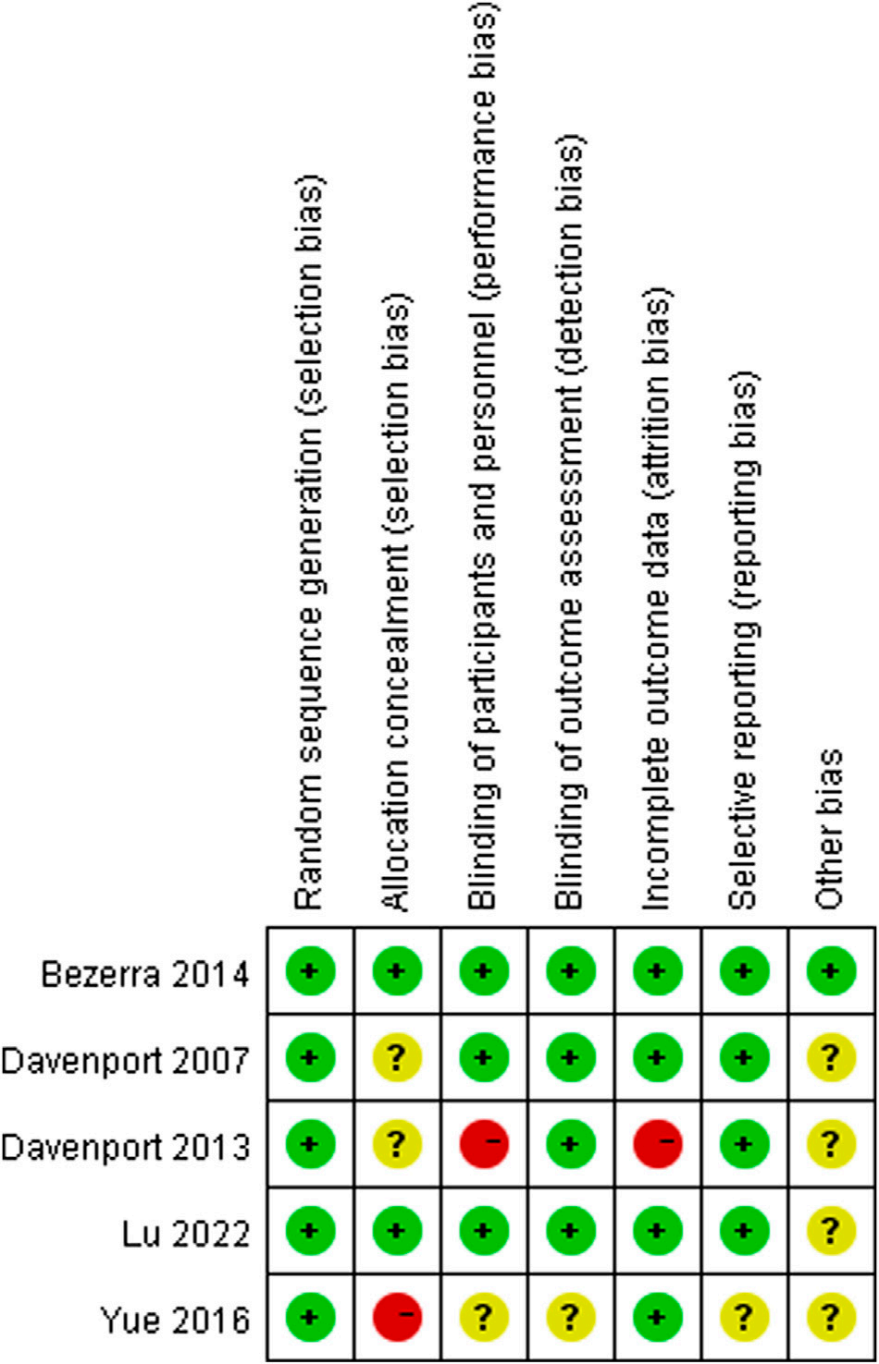
Assessment of risk of bias. Low risk of bias in green; moderate risk of bias in yellow; high risk of bias in red.

The NOS scores of the included cohort studies are shown in Supplementary Data Sheet S1 and Supplementary Table S3; three studies obtained 7 points and the remaining three studies obtained 8 points. The results of quality assessments for the included RCTs are summarized in Figure 2. Among these articles, three RCTs showed low (+) or moderate (?) risk of bias. However, two RCTs were regarded as high-risk (−) due to the use of non-standard blinding methods for steroid treatment, incomplete outcome data, and irrational patient allocation.

### Jaundice clearance rate

#### Jaundice clearance rate at the 6-month follow-up

**FIGURE 3:**
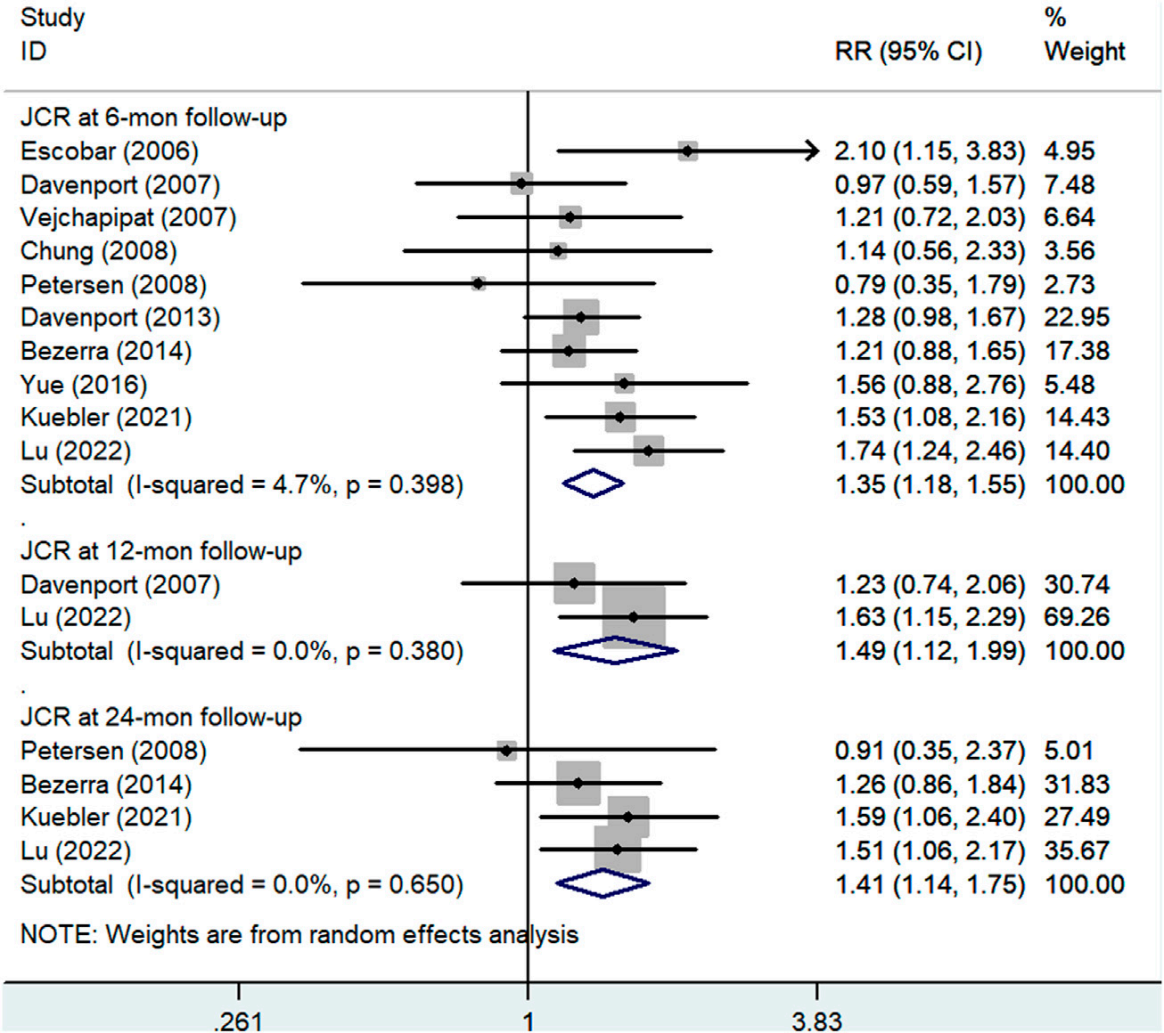
Forest plot for JCR at 6, 12, and 24 months of steroid vs. non-steroid group. JCR, jaundice clearance rate.

Figure 3 shows the results of pooled associations between steroid treatment and JCR at 6 months. Ten studies (Escobar et al., 2006; Davenport et al., 2007; Vejchapipat et al., 2007; Chung et al., 2008; Petersen et al., 2008; Davenport et al., 2013; Bezerra et al., 2014; Yue et al., 2016; Kuebler et al., 2021; Lu et al., 2022), including 507 patients in the experimental group and 497 patients in the control group, reported JCR at the 6-month follow-up. The results indicated that BA patients who received postoperative steroid therapy had significantly higher JCR at the 6-month follow-up than those who did not. The pooled RR of these ten articles was 1.35 (95% CI: 1.18–1.55; *p* < 0.001) with low heterogeneity (I^2^ = 4.7%; *p* = 0.398) using the random effects model.

**FIGURE 4:**
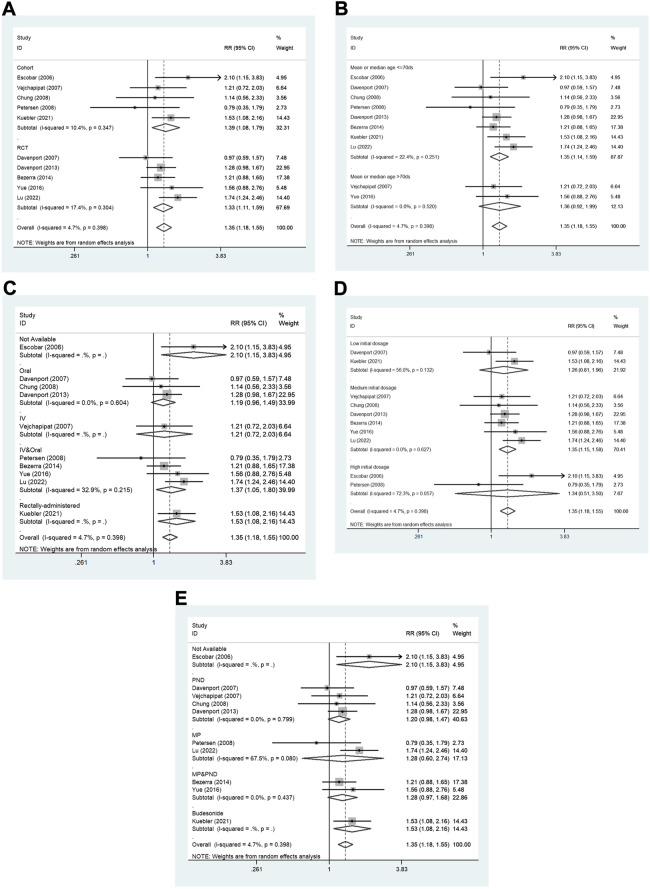
The subgroup analyses for the assessment of JCR at 6 months. **(A)** Study design. **(B)** Age at KP. **(C)** Administration method. **(D)** Initial dosage. **(E)** Steroid type. JCR, jaundice clearance rate; IV, intravenous; MP, methylprednisolone; PND, prednisone. RCT, randomized controlled trial.

Furthermore, the results of the subgroup analyses are shown in Figure 4 including Figures 4A–E,. In the subgroup analysis stratified by study design (Figure 4A), the pooled RR of cohort studies was 1.39 (95% CI: 1.08–1.79; *p* = 0.01), and the pooled RR of RCTs was 1.33 (95% CI: 1.11–1.59; *p* = 0.002). Hence, an association between steroid therapy and higher JCR at 6 months was found regardless of the study design. However, this association differed according to the other four subgroup variables. In the subgroup analysis based on age at KP (Figure 4B), the pooled RR of the population whose mean or median age was ≤70 days was 1.35 (95% CI: 1.14–1.59; *p* < 0.001), but the pooled RR of the population whose mean or median age was >70 days was 1.36 (95% CI: 0.92–1.99; *p* = 0.119). In the subgroup analysis based on administration method (Figure 4C), the pooled RR of IV followed by oral was 1.37 (95% CI: 1.05–1.80; *p* = 0.022), and the pooled RR of oral only was 1.19 (95% CI: 0.96–1.49; *p* = 0.466). In the subgroup analysis based on initial dosage (Figure 4D), the pooled RR of medium initial dosage was 1.35 (95% CI: 1.15–1.58; *p* < 0.001), the pooled RR of low initial dosage was 1.26 (95% CI: 0.81–1.96; *p* = 0.312), and the pooled RR of high initial dosage was 1.34 (95% CI, 0.51–3.50; *p* = 0.551). In the subgroup analysis based on steroid type (Figure 4E), the pooled RR of prednisone was 1.20 (95% CI: 0.98–1.47; *p* = 0.084), the pooled RR of methylprednisolone was 1.28 (95% CI: 0.60–2.74; *p* = 0.516), and the pooled RR of methylprednisolone followed by prednisone was 1.28 (95% CI, 0.97–1.68; *p* = 0.078). In conclusion, statistically significant associations were found in only specific subgroups, such as mean or median age at KP ≤ 70 days, IV followed by oral method, and medium initial dosage.

#### Jaundice clearance rate at the 12-month follow-up

Two articles (Davenport et al., 2007; Lu et al., 2022), including 132 cases in the experimental group and 137 cases in the control group, reported JCR at 12 months. The pooled RR of the analysis was 1.49 (95% CI: 1.12–1.99; *p* = 0.006) using the random effects model. This result suggested that the patients who received steroid therapy achieved a higher JCR at the 12-month follow-up (Figure 3).

#### Jaundice clearance rate at the 24-month follow-up

As shown in Figure 3, four articles (Petersen et al., 2008; Bezerra et al., 2014; Kuebler et al., 2021; Lu et al., 2022), including 283 patients in the experimental group and 280 patients in the control group, reported JCR at the 24-month follow-up. The pooled RR of the analysis was 1.41 (95% CI: 1.14–1.75; *p* = 0.002) using the random effects model. This result also indicated that postoperative steroid treatment was significantly associated with higher JCR at the 24-month follow-up.

### Native liver survival rate

#### Native liver survival rate at the 6-month follow-up

**FIGURE 5:**
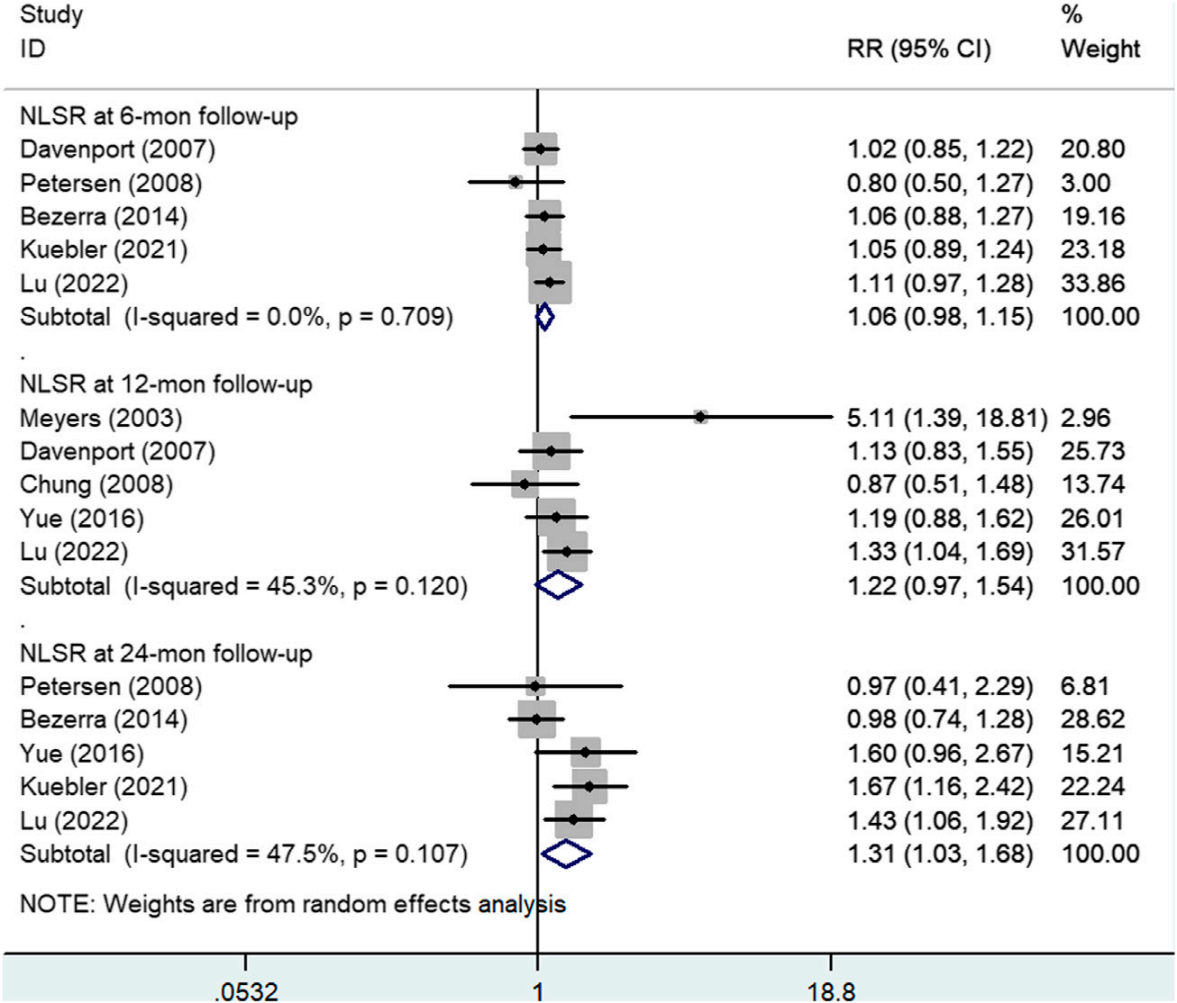
Forest plot for NLSR at 6, 12, and 24 months of steroid vs. non-steroid group. NLSR, native liver survival rate.

Figure 5 shows the results of the pooled associations between steroid treatment and NLSRs at 6 months. A total of five studies (Davenport et al., 2007; Petersen et al., 2008; Bezerra et al., 2014; Kuebler et al., 2021; Lu et al., 2022), including 317 experimental patients and 317 control patients, reported NLSR at 6 months after KP. The result showed that there was no significant difference between patients who received steroid treatment and those who did not (RR, 1.06; 95% CI: 0.98–1.15; *p* = 0.17).

#### Native liver survival rate at the 12-month follow-up

Five articles (Meyers et al., 2003; Davenport et al., 2007; Chung et al., 2008; Yue et al., 2016; Lu et al., 2022), including 220 experimental participants and 187 control participants, reported NLSR at the 12-month follow-up. Random effects model analysis yielded a pooled RR of 1.22 (95% CI: 0.97–1.54), and the difference was not statistically significant (*p* = 0.095), suggesting that patients who received steroids could not achieved higher NLSRs at 12 months after KP than those in the control group (Figure 5).

**FIGURE 6:**
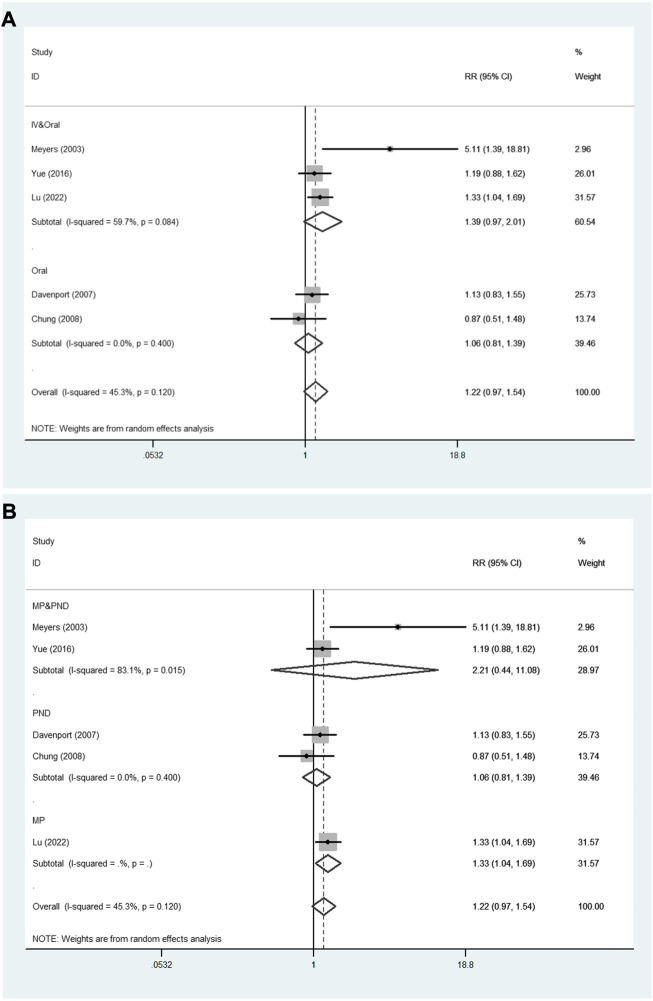
The subgroup analyses for the assessment of NLSR at 12 months. **(A)** Administration method. **(B)** Steroid type. NLSR, native liver survival rate; IV, intravenous; MP, methylprednisolone; PND, prednisone.

In addition, Figure 6 shows the results of subgroup analyses based on administration method and steroid type. In the subgroup analysis based on administration method (Figure 6A), the pooled RR of oral method was 1.06 (95% CI: 0.81–1.39; *p* = 0.673), and the pooled RR of IV followed by oral method was 1.39 (95% CI: 0.97–2.01; *p* = 0.076). Subgroup analysis according to steroid type (Figure 6B) showed that the pooled RR of methylprednisolone followed by prednisone was 2.21 (95% CI: 0.44–11.08; *p* = 0.334). The pooled RR of prednisone was 1.06 (95% CI: 0.81–1.39; *p* = 0.673).

#### Native liver survival rate at the 24-month follow-up

Five studies (Petersen et al., 2008; Bezerra et al., 2014; Yue et al., 2016; Kuebler et al., 2021; Lu et al., 2022) in which there was a total of 344 experimental patients and 300 control patients provided data on NLSR at the 24-month follow-up, as shown in Figure 5. A significant difference was observed between the study and control groups using a random effects model (RR: 1.31; 95% CI: 1.03–1.68; *p* = 0.028). It was concluded that steroid therapy can significantly improve NLSR at 24 months after KP.

### Incidence of postoperative cholangitis

**FIGURE 7:**
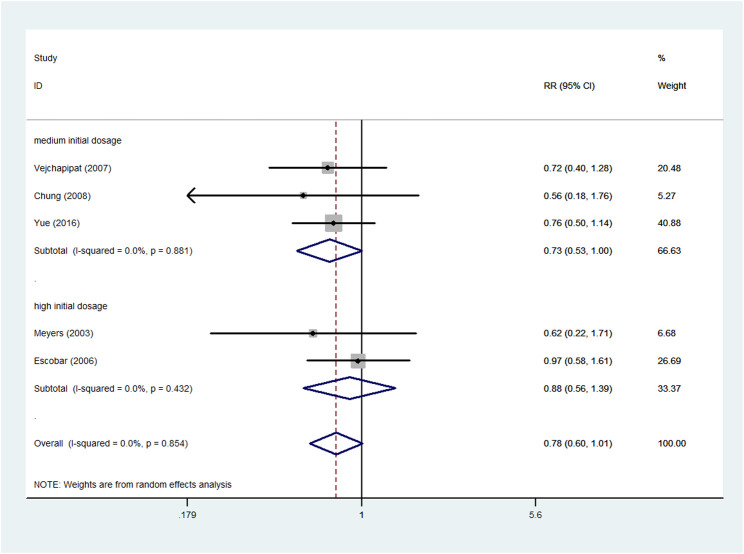
Forest plot for postoperative cholangitis rate of steroid vs. non-steroid group, and the subgroup analysis for initial dosage.

Five studies (Meyers et al., 2003; Escobar et al., 2006; Vejchapipat et al., 2007; Chung et al., 2008; Yue et al., 2016), involving 142 experimental patients and 92 control patients, reported the incidence of postoperative cholangitis. The pooled RR of the five articles was 0.78 (95% CI: 0.60–1.01; *p* = 0.058), without heterogeneity (I^2^ = 0.0%; *p* = 0.854). A random effects model was chosen for the analysis, and the result signified that adjuvant steroid therapy was not associated with the incidence of postoperative cholangitis (Figure 7).

Furthermore, the different initial dosages were analyzed in the subgroups (Figure 7). No significant difference was noted in the high initial dosage subgroup. The pooled RR of high initial dosage was 0.88 (95% CI: 0.56–1.39; *p* = 0.6), and the pooled RR of medium initial dosage was 0.73 (95% CI: 0.53–1.00; *p* = 0.051).

### Sensitivity analysis and publication bias

**FIGURE 8:**
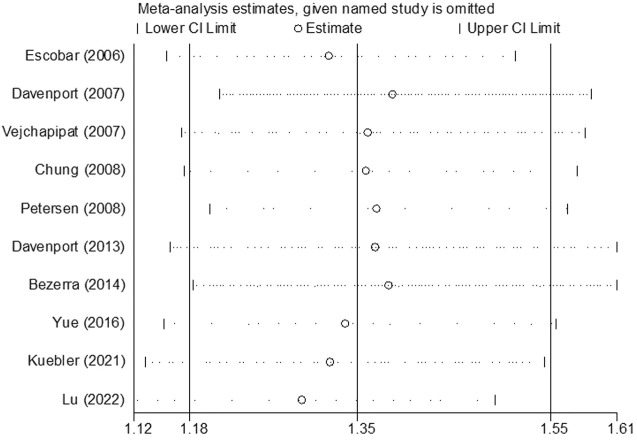
The result of sensitivity analysis regarding the assessment of JCR at 6 months. JCR, jaundice clearance rate.

**FIGURE 9:**
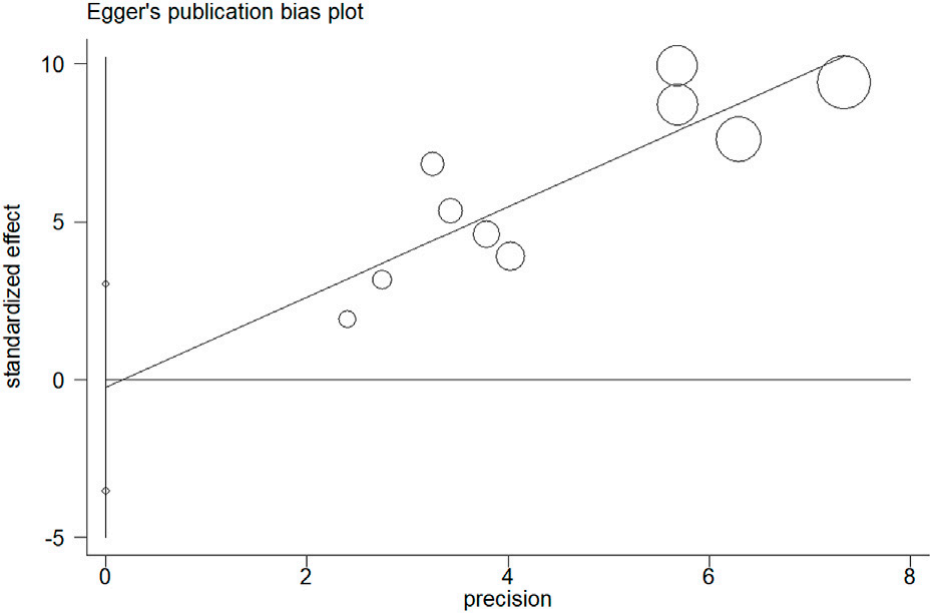
The result of publication bias for the assessment of JCR at 6 months by Egger test. JCR, jaundice clearance rate.

The results of sensitivity analysis for assessment regarding JCR at 6 months are shown in Figure 8. It indicated that the final results cannot be substantially changed by any one study. Further, the results of sensitivity analyses for other assessments, including those of JCR at the 12/24-month follow-up, NSLR at the 6/12/24-month follow-up, and the incidence of cholangitis also proved the stability of analyses (Supplementary Figures S1–S6). In addition, Figure 9 shows the result of publication bias for 10 articles regarding the assessment of JCR at 6 months. This indicated that the publication bias shown by Egger test was not significant (*p* = 0.866). However, publication bias related to JCR at 12/24 months, NLSR at 6/12/24 months, and postoperative cholangitis was not evaluated due to the limited number of included articles.

## Discussion

As previously reported (De Bosscher et al., 2000; Dillon et al., 2001; Sokol, 2007), the potential benefits of glucocorticoid therapy for BA patients include the following, (I) increases apical sodium-independent bile acid transporters, which regulate bile acid metabolism and reduce bile acid production, (II) suppresses immune function to delay progressive hepatic fibrosis, and (III) represses related inflammation gene transcription. The present meta-analysis concluded that postoperative adjuvant steroid therapy was significantly associated with bile flow and long-term native liver survival, which is consistent with the pharmacological mechanism of steroids in BA patients (De Bosscher et al., 2000; Dillon et al., 2001; Sokol, 2007). Based on the possible systemic differences of enrolled studies, we chosen the random effects model to rather than fixed model to ensure conservative effect estimates, though the heterogeneity of all studies was low (*I* ^*2*^ < 50%). Further, we compared the results of the random effects model and the fixed effects model and found that there was no difference between the two results of the most outcome indicators. In addition, the results of the sensitivity analysis and subgroup analysis of study design indicated that the conclusion has good stability and reliability.

Furthermore, previous studies indicated that age at KP and various steroid regimens were potential factors associated with clinical outcomes in patients with BA (Davenport et al., 2007; Petersen et al., 2008; Davenport et al., 2013; Okubo et al., 2020). Hence, this study performed subgroup analyses based on these variables. Significantly, subgroup analyses demonstrated that the advantages of steroid therapy seemed to only be found in particular populations and particular administration method and initial dosage. Thus far, however, there was no study has confirmed that age at KP could affect the efficacy of steroids. The present meta-analysis indicated that patients who underwent earlier KP (≤70 days) might be more sensitive to postoperative steroid therapy. This result may be due to the gradual aggravation of biliary obstruction and liver damage caused by late surgery, which could not be reversed by the use of steroids. Further studies are needed to confirm this hypothesis. In addition, there are many studies regarding the potential influence of various steroid regimens in BA patients, but there are discrepancies in their results. A study by Dong et al. reported that the IV followed by oral method of steroid administration had a superior effect on JCR and cholangitis compared to the oral method (Dong et al., 2015). A study (Kuebler et al., 2021) recommended rectal steroid administration, which showed a favorable effect on JCR at 6 months. The choice of the initial dosage of the steroid regimen is also inconsistent and disputable. A study by the Japanese Biliary Atresia Society reported that there was no significant difference between low (2 mg/kg/d) and medium (4 mg/kg/d) initial dosages in the effect on jaundice clearance (Japanese Biliary Atresia Society et al., 2013). However, a study by Dong et al. suggested that high dosage prednisone was more efficacious than lower dosage prednisone, attributing to a more stable blood concentration (Dong et al., 2013). Regarding steroid type, Zhang et al. reported that methylprednisolone was better than dexamethasone (Zhang M et al., 2017). However, this association was not found in the present study.

Several studies have reported complications that might be associated with steroid use, including wound infection, growth retardation, gastrointestinal hemorrhage, liver abscesses, and anastomotic leakage (Japanese Biliary Atresia Society et al., 2013; Bezerra et al., 2014). However, some studies (Escobar et al., 2006; Lu et al., 2022) suggest that there is no evidence that steroids increase the risk of serious complications. Even in the situations in which complications were observed, it was often difficult to determine whether the complications were associated with steroid use. This is because some complications may be related to the surgical procedure or may have been masked by effects of other adjuvant treatments (ursodeoxycholic acid, antibiotics, and fat-soluble vitamin). We were unable to perform further analysis of our pooled data; therefore, further studies regarding the safety of steroid therapy are necessary.

The present meta-analysis had several strengths. First, this is the first meta-analysis to confirm the presence of a significant association between postoperative steroid use and better clinical outcomes, especially long-term outcomes. Second, with the increase in the included data, this meta-analysis analyzed the effect of adjuvant steroid therapy on the rate of occurrence of postoperative cholangitis. Third, subgroup analyses according to possible influencing factors, including administration method, initial dosage, and steroid type, were conducted in the present analysis. This meta-analysis is also the first to confirm the association between the efficacies of steroid and steroid regimens. These results may be useful in clinical practice and for future research.

Our meta-analysis had some limitations. First, we included cohort studies and RCTs. However, the subgroup analysis indicated that the results were not appreciably modified by the study design. Furthermore, in the included studies, two recent large-sample studies (Kuebler et al., 2021; Lu et al., 2022) played an important statistical role that could have greatly influenced the final result. Consequently, we used a random-effects model to balance effect size weights. Second, in these pooled studies, the definition of native liver survival rate was inconsistent. Some studies (Petersen et al., 2008; Bezerra et al., 2014; Kuebler et al., 2021) defined the indicator as the native liver survival with normal bilirubin levels, but other studies (Davenport et al., 2007; Lu et al., 2022) did not did not include a clear definition of NLSR. Thus, in the future, NLSR should be defined as native liver survival with normal bilirubin level. Third, because of the data limitation, our subgroup analyses were only involved three outcome indicators, including JCR at 6 months, NLSR at 12 months, and postoperative cholangitis rate. Further studies on the potential influence of various steroid regimens on more outcome indicators are needed. Fourth, we did not analyze the association between steroids and operative complications or other adjuvant treatments because of the limited data in the included articles. Therefore, further studies regarding this are also required.

## Conclusion

In conclusion, this meta-analysis evaluated the effect of postoperative adjuvant steroid therapy on clinical outcomes in patients with BA, and included subgroup analyses according to age at KP and steroid regimens. The results show that postoperative adjuvant steroid therapy could significantly improve JCR and long-term NLSR, except for short-term NLSR and postoperative cholangitis. The superiority of steroid use was more remarkable in patients aged ≤70 days at KP. Significantly, the results demonstrate that the most reliable efficiency of steroids appear to be associated with the following factors: The IV followed by oral method and medium initial dosage. Although further studies are warranted, the present meta-analysis clarified the favorable effect of postoperative adjuvant steroid therapy and suggested that the efficiency of steroid might be significantly affected by age at KP and various steroid regimens.

## Data Availability

The original contributions presented in the study are included in the article/Supplementary Material, further inquiries can be directed to the corresponding author.

## Supplementary material

Supplementary Figure S1 The result of sensitivity analysis regarding JCR at 12 months.

Supplementary Figure S2 The result of sensitivity analysis regarding JCR at 24 months.

Supplementary Figure S3 The result of sensitivity analysis regarding NLSR at 6 months.

Supplementary Figure S4 The result of sensitivity analysis regarding NLSR at 12 months.

Supplementary Figure S5 The result of sensitivity analysis regarding NLSR at 24 months.

Supplementary Figure S6 The result of sensitivity analysis regarding postoperative cholangitis.

## Abbreviations

BA, biliary atresia; CI, confidence interval; IV, intravenous; JCR, jaundice clearance rate; KP, Kasai portoenterostomy; NLSR, native liver survival rate; RR, risk ratio

**Declaration of Competing Interest:** All authors have no conflict of interest to disclose.
