# Supplementary figures and images for: Effects of postoperative adjuvant steroid therapy on the outcomes of biliary atresia: A systematic review and updated meta-analysis
Source: Front Pharmacol. 2022 Sep 14;13:956093. doi: 10.3389/fphar.2022.956093 (PMC9516003; doi:10.3389/fphar.2022.956093)

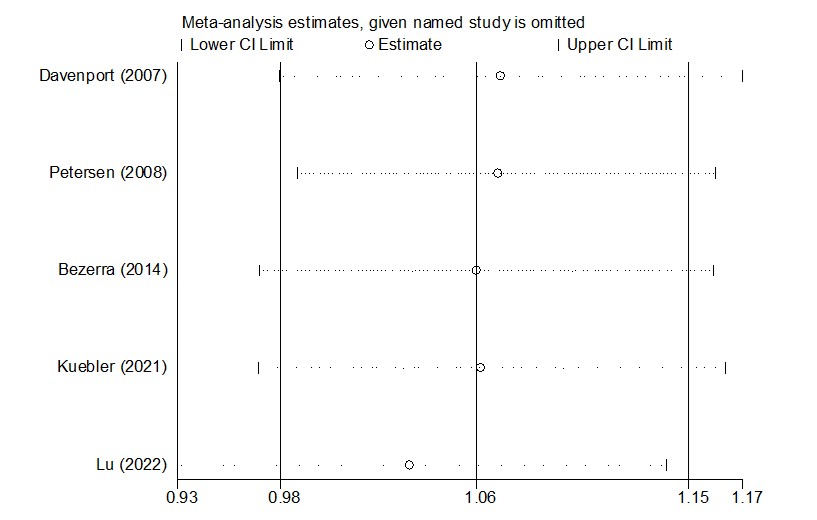

Supplement: Supplementary file 1 [file Image3.JPEG]

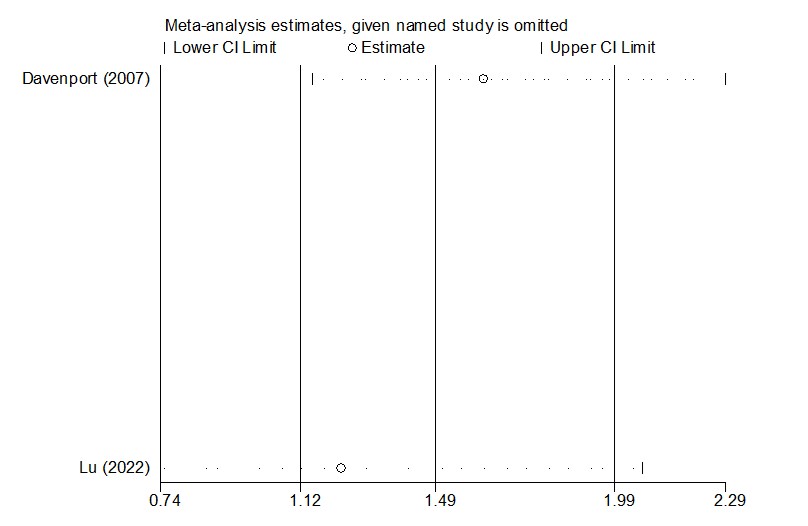

Supplement: Supplementary file 2 [file Image1.JPEG]

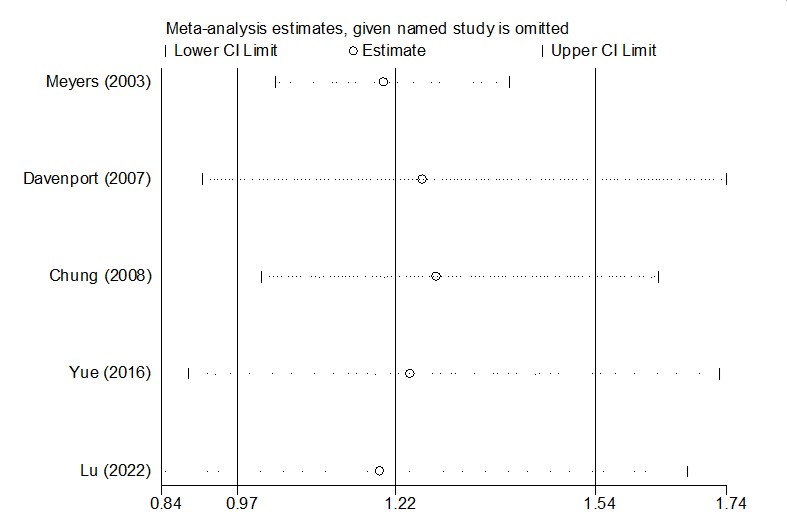

Supplement: Supplementary file 3 [file Image4.JPEG]

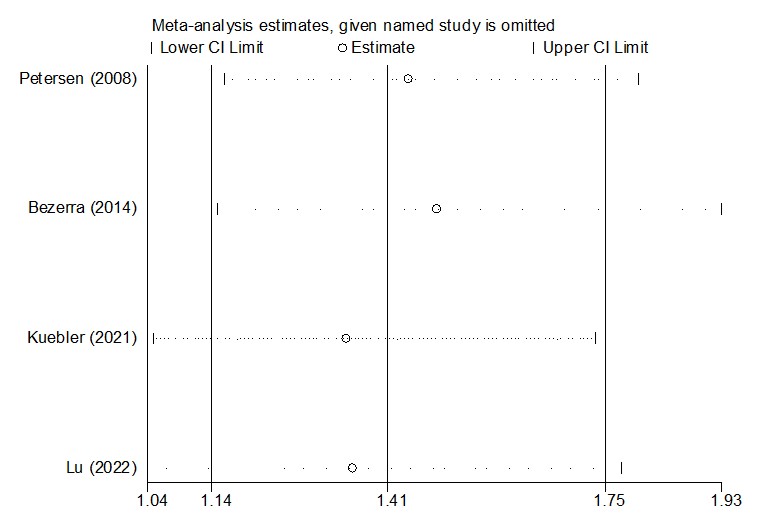

Supplement: Supplementary file 4 [file Image2.JPEG]

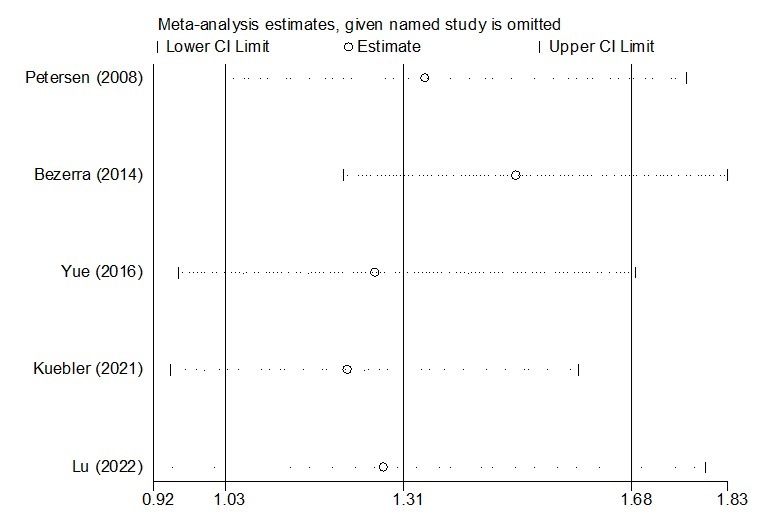

Supplement: Supplementary file 5 [file Image5.JPEG]

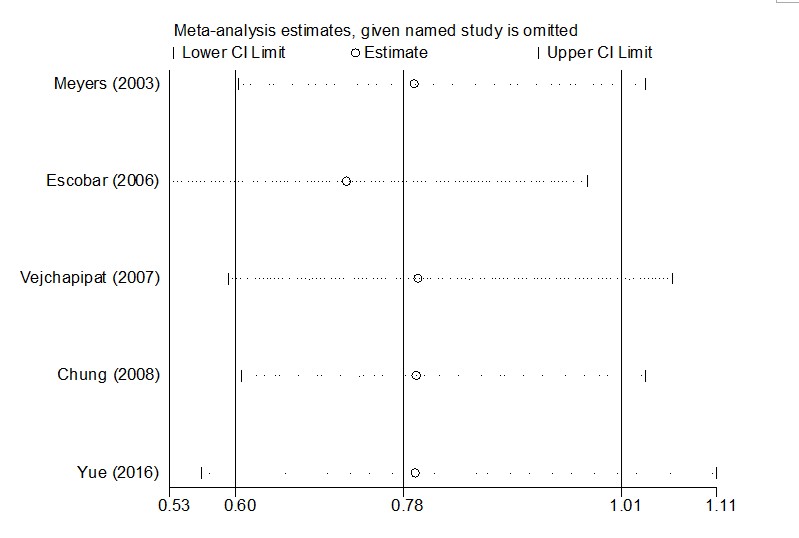

Supplement: Supplementary file 6 [file Image6.JPEG]
